# Biomarkers of Inflammation Increase with Tau and Neurodegeneration but not with Amyloid-β in a Heterogenous Clinical Cohort

**DOI:** 10.3233/JAD-220523

**Published:** 2022-10-11

**Authors:** Sofia Michopoulou, Angus Prosser, Christopher Kipps, John Dickson, Matthew Guy, Jessica Teeling

**Affiliations:** a Imaging Physics, University Hospital Southampton, Southampton, UK; b Faculty of Medicine, University of Southampton, Southampton, UK; c Institute of Nuclear Medicine, University College London Hospitals, London, UK; d School of Biological Sciences, University of Southampton, Southampton, UK; e Interdisciplinary Dementia and Imaging Centre (iDeAC), Southampton, UK

**Keywords:** Amyloid-β, dementia, inflammation, tau protein

## Abstract

**Background:**

Neuroinflammation is an integral part of Alzheimer’s disease (AD) pathology. Inflammatory mediators can exacerbate the production of amyloid-β (Aβ), the propagation of tau pathology and neuronal loss.

**Objective:**

To evaluate the relationship between inflammation markers and established markers of AD in a mixed memory clinic cohort.

**Methods:**

105 cerebrospinal fluid (CSF) samples from a clinical cohort under investigation for cognitive complaints were analyzed. Levels of Aβ_42_, total tau, and phosphorylated tau were measured as part of the clinical pathway. Analysis of inflammation markers in CSF samples was performed using multiplex immune assays. Participants were grouped according to their Aβ, tau, and neurodegeneration status and the Paris-Lille-Montpellier (PLM) scale was used to assess the likelihood of AD.

**Results:**

From 102 inflammatory markers analyzed, 19 and 23 markers were significantly associated with CSF total tau and phosphorylated tau levels respectively (*p* < 0.001), while none were associated with Aβ_42_. The CSF concentrations of 4 inflammation markers were markedly elevated with increasing PLM class indicating increased likelihood of AD (*p* < 0.001). Adenosine deaminase, an enzyme involved in sleep homeostasis, was the single best predictor of high likelihood of AD (AUROC 0.788). Functional pathway analysis demonstrated a widespread role for inflammation in neurodegeneration, with certain pathways explaining over 30% of the variability in tau values.

**Conclusion:**

CSF inflammation markers increase significantly with tau and neurodegeneration, but not with Aβ in this mixed memory clinic cohort. Thus, such markers could become useful for the clinical diagnosis of neurodegenerative disorders alongside the established Aβ and tau measures.

## INTRODUCTION

Alzheimer’s disease (AD) is a chronic neurodegenerative disease that starts decades before the presentation of symptoms. Its histopathological hallmarks include the accumulation of amyloid-β (Aβ) plaques, the formation of neurofibrillary tangles from aggregation of pathogenic hyperphosphorylated tau protein and the activation of microglia and astrocytes [1].

Measurements of Aβ_42_, phosphorylated tau (ptau), and total tau in cerebrospinal fluid (CSF) provide sensitive and specific markers of disease [2]. These three CSF markers can be used to group patients according to their status of Aβ (A), tau (T), and neurodegeneration (N), using the ATN framework which defines the AD spectrum in terms of biomarkers focusing on the biology of AD [3]. Additionally, the PLM scale relies on the same CSF markers to provide a classification of the likelihood of a patient having AD in the following categories, Class 0: very low (<10%), Class 1: low (<25%), Class 2: high (>75%), and Class 3: very high (>90%) for positive AD [4]. The ATN framework and PLM scale are useful in diagnosing AD and other neurodegenerative diseases but lack insight into other factors that contribute to the onset and/or progression of disease.

Neuroinflammation is an integral part of AD pathology [5], and a recent review article by Webers et al. concluded that “there is now substantial evidence that neuroinflammation contributes to the progression of AD” [6]. Inflammatory mediators exacerbate the production of Aβ, the propagation of tau pathology and neuronal loss [7]. Several recent studies have identified increased CSF levels of a wide range of inflammatory markers including in patients with AD in well controlled research cohorts [8–10]. These markers may provide complementary information to Aβ and tau biomarkers for AD diagnosis and prognosis. Better understanding of inflammation pathways in AD may also help identify new therapeutic targets and facilitate monitoring the efficacy of such therapies [11].

The inflammatory response is highly dynamic and variable and a challenge in biomarker research is to identify a reliable marker, or composite marker, that accompanies the current framework of diagnosis and prognosis of AD in real-world cohorts. To assess their viability for clinical application, we aimed to evaluate if changes in CSF inflammation markers are robust enough to be detectable in a heterogenous clinical population without exclusion criteria.

## MATERIALS AND METHODS

### Patients and data

Participants with cognitive complaints, suspected to be due to underlying neurodegenerative pathology were referred to the Wessex Neurology Clinic at University Hospital Southampton NHS Foundation Trust between 2014 and 2021, where they underwent diagnostic lumbar puncture. Participants or their next of kin provided written informed consent at the time of the lumbar puncture and gave permission for storage of excess CSF. Inclusion criteria for the present study were: 1) referral for cognitive complaints with query dementia; and 2) availability of AD biomarker results and CSF sample for inflammation analysis. No exclusion criteria applied, in order to evaluate the use of inflammation markers in a mixed memory clinic cohort. The study was approved by the Research Ethics Committee (REC 20/NW/0222).

To assess the relationship between Aβ, tau, and neurodegeneration and levels of inflammatory markers were measured in 105 participants CSF samples using multiplex assays, as outlined in the following sections. Participants were classified as positive or negative for Aβ (A+/–), tauopathy (T+/–), and neurodegeneration (N+/–) as outlined in the NIA-AA framework using the threshold values for Aβ_42_, ptau, and total tau CSF concentration respectively as outlined in Table 1 [12–14].

**Table 1:** CSF concentration thresholds for ATN classification

ATN Class	CSF concentration
A+	Aβ_42_ <680 pg/ml
T+	ptau >56 pg/ml
N+	total tau >355 pg/ml

In addition to evaluating the ATN status as separate markers of disease in this study, the resulting classifications were used to categorize participants with the PLM framework in order to assess the combined likelihood of AD. The PLM class was produced by counting the number of pathological markers according to these predefined thresholds; i.e. PLM Class 0 for no pathological markers, PLM Class 1 for one pathological marker, PLM Class 2 for two pathological markers, and PLM Class 3 if all three CSF markers of AD are outside their normal concentration levels [4]. Finally, the detected markers were grouped by their functional pathway classifications, to help identify possible underlying pathways of inflammatory changes linked to neurodegeneration.

### Sample handling and storage

A minimum of 2 ml of CSF was collected from each participant in Starstedt polypropylene tubes and centrifuged within 30 min [15]. 500μL were aliquoted in an Elkay polypropylene tube, frozen at –70°C and sent for clinical diagnostic analysis to the Neuroimmunology and CSF laboratory at UCLH [16]. The remaining sample was frozen and stored locally at –80°C. Prior to research study analysis, the samples were thawed, partitioned in various sample sizes including 80 samples and aliquoted in low-absorbance microtiter plates. Samples were refrozen and stored at –80°C. Samples in microtiter plates remained frozen until the analysis of the inflammation markers. Samples were anonymized and randomized so that researchers were blinded to each participant’s underlying condition.

### Sample analysis for clinical diagnosis

CSF Aβ_42_, total tau, and ptau levels were measured by the UKAS accredited Neuroimmunology and CSF laboratory at the National Hospital for Neurology and Neurosurgery at Queen Square London as part of normal clinical evaluation. The samples were analyzed using the INNOTEST (Fujirebio) enzyme-linked immunosorbent assays (ELISA) system until 2020, while the Lumipulse G (Fujirebio) chemiluminescent immunoassay was used thereafter. Both methods use the same antibody pair, automatic corrections are included in the Lumipulse software to retain equivalence with the ELISA where appropriate and results are consistent [17, 18].

### Sample analysis for research study

Targeted proteomic analysis with two assay methods was used to measure 102 inflammation markers from each patients’ CSF sample. The V-Plex Proinflammatory Panel 1 human kit (Mesoscale, cat no K15049D) was used to quantify the concentration of IFN-*γ*, IL-1β, IL-2, IL-4, IL-6, IL-8, IL-10, IL-12p70, IL-13, and TNF-α. This analysis is based on electrochemiluminescence immunoassay, providing high sensitivity quantification of inflammation marker concentration [19]. The Olink® Target 96 Inflammation Panel was used to measure levels of 92 inflammatory markers. The proximity extension array technology allows oligonucleotide labelled antibody probe pairs to bind to their respective target proteins in the samples and a polymerase chain reaction (PCR) reporter sequence is formed only when two antibodies are in close proximity. This sequence is then detected by quantitative PCR and measured in Normalized Protein eXpression (NPX) units, which are arbitrary units in a log2 scale produced through data normalization [20]. Only markers detected in over 90% of samples were included in the analysis, to ensure data quality by providing wide sample representation which would facilitate reproducibility across different patient cohorts. Across the different samples and markers, 60% of Mesoscale and 63% of Olink measurements were below the detection threshold. In total, 52 inflammation markers were detected above the threshold in more than 90% of samples and were included in further analysis. From these, two markers, namely IL-6 and IL-8, were present on both Olink and Mesoscale assay platforms. Their values were linearly correlated between the Olink and Mesoscale, with Pearson correlation coefficients of 0.84 for IL-6 and 0.77 for IL-8 (*p* < 0.001), showing good compatibility of results across the two types of assays.

### Statistical analysis

Correlation analysis using Spearman’s correlation coefficient was used to evaluate the relationship between the inflammation markers values and the AD biomarkers Aβ_42_, total tau, and ptau. Independent two sample *t*-tests were used to evaluate differences in inflammation markers for A+, T+, N+ versus A–, T–, N–participants. To control for multiple comparisons, False Discovery Rate (FDR) analysis was performed using the Benjamini-Hochberg method with an FDR <5% considered significant.

Finally, analysis of variance was performed to evaluate differences in inflammation markers based on the participants’ PLM class. SPSS v.27.0.1.0 (IBM 2020) and Matlab 2021a (Mathworks) were used for statistical analysis. Logistic regression analysis was performed for the different inflammation markers adjusting for patient age and sex. Results are displayed using receiver operator characteristic outlining the adjusted inflammation markers’ ability to differentiate between low and high likelihood of AD as defined by the PLM class.

### Functional pathway classification

The proteins analyzed in this study were classified according to the underlying biological process in the following groups: 1) apoptotic process, 2) cell activation involved in immune response, 3) cell adhesion, 4) cellular response to cytokine stimulus, 5) chemotaxis, 6) extracellular matrix organization, 7) inflammatory response, 8) mitogen activated protein kinase (MAPK) cascade, 9) regulation of immune response, 10) response to hypoxia, and 11) secretion. These processes were derived by Olink from public access databases including Uniprot, Human Protein Atlas, Gene Ontology and DisGeNET [21–25]. Most proteins are involved in multiple pathways, and these are outlined in the Supplementary Material.

Linear regression models were used to assess how well proteins in each pathway classification can predict the values of Aβ_42_, ptau, and total tau, as a means of evaluating the role of these functional pathways in AD.

## RESULTS

In this mixed memory clinic cohort, the mean age of participants was 67 years (range 35 to 88), and 40% were females. Table 2 outlines the number of participants in each group, their demographics, and the corresponding mean CSF concentrations of the AD biomarkers. Seven participants had no recorded measurement of total tau. The names, abbreviations, and functional pathway classifications for these markers are provided in Supplementary Table 1.

**Table 2:** Participants’ demographics and Clinical A, T, and N Results

	A+	A–	T+	T–	N+	N–	PLM 0	PLM 1	PLM 2	PLM 3
Number of Participants	56	49	43	62	44	54	32	28	20	25
Age mean (std)	69 (12)	66 (11)	65 (8)	70 (13)	68 (13)	69 (10)	67 (11)	70 (11)	69 (15)	66 (9)
Sex (F/M)	22/34	21/28	20/23	23/39	22/22	19/35	10/22	14/14	7/13	12/13
Aβ_42_ (pg/ml)	503	1080	646	860	701	861	1041	779	759	504
ptau (pg/ml)	75.1	41.7	96.4	34.6	81.1	38.7	34.7	38.1	61.5	106.4
total tau (pg/ml)	647	347	815	309	814	244	250	335	474	1009

### Association of inflammation markers to ad biomarker values in CSF

Table 3 shows the inflammatory markers correlation to age, Aβ_42_, ptau, and total tau concentrations in CSF. Two markers demonstrated significantly positive correlation with participant age. No inflammation markers showed significant correlation with Aβ_42_; however, there were 23 inflammation markers that correlated significantly with ptau and 19 inflammatory markers that correlated significantly with total tau levels in CSF.

**Table 3:** Spearman’s correlation of inflammation markers values to participant age and the concentration of AD biomarkers

Inflammation	Age	Aβ_42_	ptau	total tau
Marker
4E-BP1	0.102	–0.043	0.368^*^	0.364^*^
ADA	–0.068	0.026	0.600^*^	0.619^*^
CCL11	0.242	–0.004	0.042	0.021
CCL19	–0.13	0.028	0.225	0.047
CCL23	0.260	–0.104	0.176	0.137
CCL25	0.207	–0.017	0.026	–0.012
CCL3	0.290	–0.157	0.238	0.262
CCL4	0.178	0.003	0.115	0.132
CD40	0.082	0.115	0.388^*^	0.389^*^
CD5	–0.12	0.045	0.306	0.167
CD8A	–0.047	0.068	0.205	0.188
CDCP1	0.183	0.038	0.321^*^	0.322^*^
CSF-1	0.065	0.057	0.466^*^	0.415^*^
CST5	0.079	0.141	0.075	0.098
CX3CL1	–0.058	0.086	0.492^*^	0.432^*^
CXCL1	0.082	0.02	–0.038	0.069
CXCL10	–0.05	0.018	0.191	0.042
CXCL11	–0.052	0.012	0.203	0.082
CXCL5	0.078	0.02	0.194	0.065
CXCL6	–0.038	–0.045	0.091	–0.027
CXCL9	0.349^*^	–0.091	0.09	0.078
DNER	–0.009	0.092	0.433^*^	0.371^*^
FGF-19	0.048	0.077	0.323^*^	.242
FGF-5	–0.137	0.185	0.517^*^	0.470^*^
Flt3L	0.253	–0.004	0.341^*^	0.339^*^
HGF	0.119	–0.053	0.504^*^	0.458^*^
IL-10RB	0.095	0.047	0.399^*^	0.336^*^
IL-12B	–0.012	0.031	0.321^*^	0.198
IL18	0.089	0.232	0.118	0.108
IL-18R1	0.205	–0.208	0.1	0.202
IL-1b	0.156	–0.175	0.144	0.135
IL-2	0.193	–0.097	0.139	0.147
IL-6	0.111	–0.062	–0.058	0.084
IL-8	0.148	–0.087	0.124	0.148
LAP TGF-beta-1	0.144	0.085	0.284	0.189
LIF-R	–0.044	0.122	0.484^*^	0.439^*^
MCP-1	0.386^*^	–0.16	0.021	0.075
MCP-2	0.018	–0.072	0.091	0.027
MCP-4	0.204	–0.068	0.173	0.055
MMP-1	0.107	–0.01	0.250	0.259
MMP-10	0.024	–0.111	0.408^*^	0.368^*^
OPG	0.285	–0.078	0.219	0.184
PD-L1	–0.054	0.135	0.536^*^	0.462^*^
SCF	0.148	0.072	0.453^*^	0.413^*^
TGF-alpha	–0.045	0.038	0.473^*^	0.430^*^
TNFB	0.005	0	0.289	0.261
TNFRSF9	0.112	–0.008	0.350^*^	0.283
TNFSF14	–0.06	–0.036	0.303	0.169
TRAIL	0.017	0.016	0.387^*^	0.276
TWEAK	–0.041	0.102	0.616^*^	0.520^*^
uPA	0.017	–0.026	0.460^*^	0.411^*^
VEGFA	0.041	0.117	0.466^*^	0.405^*^

^*^indicates a significant correlation at *p* < 0.001, all significant correlations were positive indicating increase in inflammation with increasing AD biomarker values.

### Inflammation markers increase in patients positive for tau and neurodegeneration

Participants were grouped as positive vs negative for amyloid, tau, and neurodegenerations using the predefined thresholds for Aβ_42_, ptau, and total tau respectively as outlined in Table 1. Beyond the correlation of continuous values of inflammation versus AD biomarkers outlined on Table 3, we also analyzed grouped responses for AD biomarkers, with values above versus below the thresholds outlined in Table 1. The results of this univariate analysis of inflammation markers for participants who are positive vs negative for each of the three AD biomarkers are outlined in Table 4. Following FDR correction for multiple comparisons with a 5% threshold, none of the inflammation markers was significantly different for A+ versus A–participants. T+ participants, as defined via the ptau threshold, where found to have significantly higher levels of 25 different markers of inflammation. Finally, N+ participants, as defined by the total tau threshold, were found to have significantly higher levels of 21 different markers of inflammation.

**Table 4:** Univariate analysis of significant differences in inflammation markers for participants with positive vs negative amyloid, tau and neurodegeneration status

Inflammation Marker	amyloid-beta (Aβ_42_)		tau (ptau)		neurodegeneration (total tau)	
	*p*	FDR%	*p*	FDR%	*p*	FDR%
4E-BP1	0.05	31%	0.10	18%	0.00	**1**%
ADA	0.38	86%	0.00	**0**%	0.00	**0**%
CCL11	0.35	86%	0.72	75%	0.88	90%
CCL19	0.81	94%	0.13	20%	0.14	22%
CCL23	0.05	31%	0.13	20%	0.07	12%
CCL25	0.86	94%	0.92	94%	0.81	87%
CCL3	0.01	31%	0.05	10%	0.03	7%
CCL4	0.50	91%	0.29	38%	0.33	43%
CD40	0.80	94%	0.00	**1% **	0.00	**0% **
CD5	0.91	94%	0.02	**4% **	0.13	20%
CD8A	0.64	94%	0.10	18%	0.04	8%
CDCP1	0.86	94%	0.02	**4% **	0.04	8%
CSF-1	0.40	87%	0.00	**0% **	0.00	**0% **
CST5	0.45	91%	0.48	56%	0.45	57%
CX3CL1	0.85	94%	0.00	**0% **	0.00	**1% **
CXCL1	0.54	91%	0.71	75%	0.20	28%
CXCL10	0.81	94%	0.22	31%	0.92	92%
CXCL11	0.85	94%	0.15	23%	0.66	75%
CXCL5	0.80	94%	0.30	39%	0.89	90%
CXCL6	0.28	79%	0.63	69%	0.84	89%
CXCL9	0.16	51%	0.59	67%	0.20	28%
DNER	0.62	94%	0.00	**0% **	0.00	**0% **
FGF-19	0.94	94%	0.00	**1% **	0.07	12%
FGF-5	0.54	91%	0.00	**0% **	0.00	**0% **
Flt3L	0.14	49%	0.02	**4% **	0.00	**1% **
HGF	0.06	31%	0.00	**0% **	0.00	**0% **
IL-10RB	0.67	94%	0.00	**0% **	0.00	**1% **
IL-12B	0.88	94%	0.01	**3% **	0.12	19%
IL-18R1	0.13	49%	0.37	46%	0.16	23%
IL-1b	0.06	31%	0.11	18%	0.05	10%
IL-2	0.10	40%	0.68	73%	0.26	34%
IL-6	0.04	31%	0.38	46%	0.55	66%
IL-8	0.01	31%	0.50	58%	0.01	**2% **
IL18	0.04	31%	0.28	38%	0.06	10%
LAP TGF-beta	0.75	94%	0.11	18%	0.62	73%
LIF-R	0.91	94%	0.00	**0% **	0.00	**1% **
MCP-1	0.06	31%	0.39	46%	0.48	59%
MCP-2	0.15	51%	0.32	42%	0.71	78%
MCP-4	0.30	79%	0.19	28%	0.67	75%
MMP-1	0.23	69%	0.02	**4% **	0.01	**3% **
MMP-10	0.07	36%	0.00	**1% **	0.00	**0% **
OPG	0.08	37%	0.05	10%	0.07	12%
PD-L1	0.61	94%	0.00	**0% **	0.00	**0% **
SCF	0.46	91%	0.00	**0% **	0.00	**0% **
TGF-alpha	0.51	91%	0.00	**0% **	0.00	**0% **
TNFB	0.93	94%	0.05	10%	0.04	8%
TNFRSF9	0.61	94%	0.01	2%	0.01	**4% **
TNFSF14	0.80	94%	0.02	5%	0.14	21%
TRAIL	0.50	91%	0.00	**1% **	0.03	7%
TWEAK	0.85	94%	0.00	**0% **	0.00	**0% **
uPA	0.37	86%	0.00	**0% **	0.00	**0% **
VEGFA	0.91	94%	0.00	**0% **	0.00	**0% **

Statistically significant difference for FDR <5% outlined in **bold**.

### Gradual increase in inflammation with increasing likelihood of AD

**Fig. 1:**
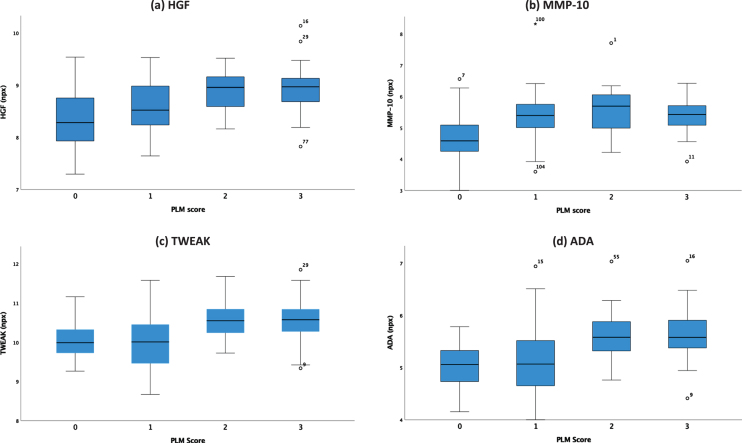
Boxplots outlining changes in inflammation marker concentration in units of normalized protein expression (npx) with PLM class. The box edges correspond to the interquartile range and the T-bars give the 95% intervals.

**Fig. 2:**
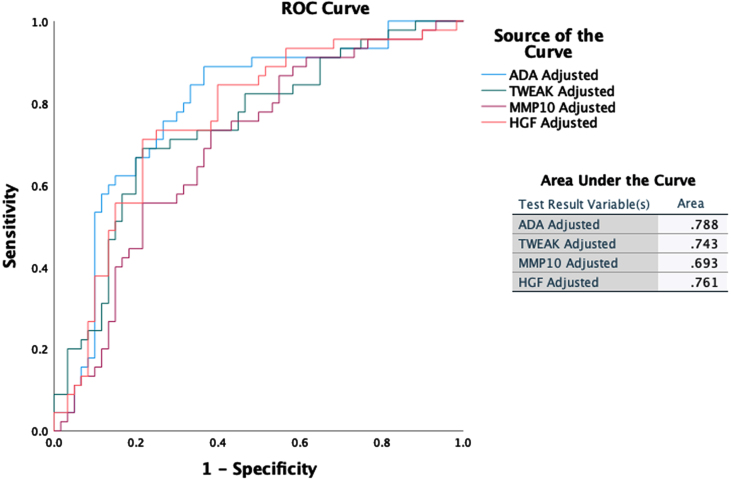
ROC curves and AUROC scores for HGF, MMP-10, ADA, and TWEAK adjusted for participant’s age and sex as independent predictors of high likelihood of AD.

Inflammation markers that increase significantly with greater likelihood of AD diagnosis as defined by increasing PLM class (ANOVA, *p* < 0.001) include: hepatocyte growth factor (HGF), matrix metalloproteinase-10 (MMP-10), tumor necrosis factor superfamily member 12 (TWEAK), and adenosine deaminase (ADA). Figure 1 outlines the changes in the values of these markers with PLM class. Figure 2 shows ROC curves outlining the ability of these four inflammation markers, following adjustment for patient age and sex, to differentiate participants with high and very high risk of AD (PLM 2 or 3) from those with very low and low risk of AD (PLM 0 or 1). ADA demonstrates the best classification capability with an area under the ROC (AUROC) score of 0.788.

### Functional pathways of inflammation in Alzheimer’s disease

**Fig. 3:**
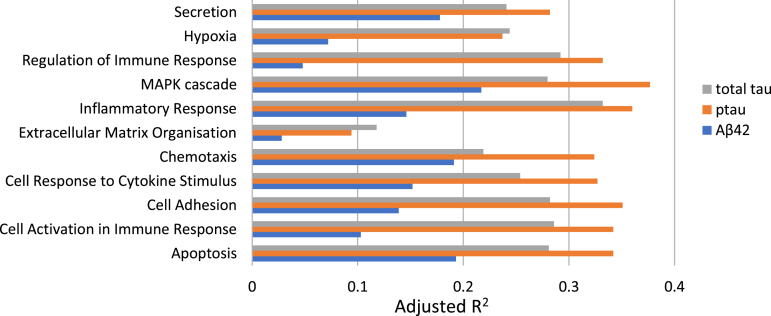
Prediction of Aβ_42_, ptau, and total tau values from inflammatory proteins across 11 functional pathways using linear regression models. The adjusted R^2^ indicates the variance of each AD biomarker explained by each inflammatory pathway.

CSF measures of inflammation were identified across all functional pathways, and measures in 11 pathways explained 7% –39% of the variance in Aβ_42_, ptau, and total tau values (outlined in the adjusted R^2^ diagram in Fig. 3).

For ptau, 10 inflammation pathways yielded predictions significantly better than expected by chance (*p* < 0.001) and explained variance of between 23% and 38%. The Extracellular Matrix Organisation pathway only explained 9% of variance here and was not significant. For total tau, 8 different pathways explained between 24% and 33% of the variance in total tau values. Here Extracellular Matrix Organisation, Chemotaxis, and Cell Response to Cytokine Stimulus pathways were not significant at the *p* < 0.001 threshold. Finally, 7–22% of the variance in Aβ_42_ values was explained by the inflammation pathways, but no pathways reached statistical significance at the *p* < 0.001 threshold. The strongest overall predictor for total tau was the Inflammatory Response pathway (R^2^ 0.33) and for ptau the MAPK Cascade pathway (R^2^ 0.38).

## DISCUSSION

Aβ is considered a central part of the pathogenesis of AD, while tau is better correlated with disease progression [26–28]. Our analysis identified positive associations between markers of inflammation and ptau and total tau values. Over 20 of the inflammation markers measured in CSF were significantly higher in T+ and/or N+ participants compared to participants who were T–and/or N–. Four inflammatory markers; namely MMP-10, HGF, ADA, and TWEAK, increased with higher likelihood of AD as expressed by the PLM class. Associations between markers of inflammation and Aβ_42_ values were not significant in our mixed memory clinic cohort. Our findings, and the association of inflammation markers with ptau and total tau but not Aβ_42_, support a primary role for inflammation in neurodegeneration, presumed due to AD, which is consistent with preclinical and clinical studies [6, 10, 29]. Our results indicate that inflammation markers may be closely associated with neurodegenerative pathways in cognitively impaired individuals, and that they may be useful as predictors of future decline in a mixed memory clinic cohort. Longitudinal studies are required to assess the value of inflammation markers in predicting the risk of dementia progression to help inform medical management and provision of care.

At the level of individual markers, multiple studies have shown that MMP-10 levels are increased in AD and frontotemporal dementia (FTD) CSF compared to healthy controls [9, 30, 31]. Matrix metallo-proteinases (MMPs) are important enzymes for the function of the blood-brain barrier, with both detrimental and beneficial effects for the host, depending on the MMP studied. MMP-10 (or stromelysin-2) plays a key role in the host response to environmental stimuli, and its expression is induced following injury, infection, or transformation. This metalloproteinase is involved in the breakdown of extracellular matrix and has been shown to have a profibrinolytic effect in an ischemic stroke model, reducing infarct size. MMP-10 also serves a protective role in acute infection by moderating the proinflammatory activity of macrophages [32, 33]. Duits et al. studied both MMPs and tissue inhibitors of matrix metalloproteinases (TIMPs) in AD. They found an increase in CSF levels of MMP-10 coupled with a decrease in TIMPs in participants with AD and concurrent cerebral microbleeds. Their findings suggest that increased levels of MMPs are associated with a more vulnerable blood-brain barrier in AD but if MMP-10 contributes to injury or repair remains to be investigated [14]. Whelan et al. measured multiple biomarkers in CSF and blood in AD and MCI patients from the Swedish BioFINDER study. Compared to cognitively normal elderly controls, both AD patients and Aβ+ MCI patients showed significant increased levels of CSF inflammatory markers, including MMP-10, correlating with cognitive performance, and cortical thickness. outlining its potential as a prognostic marker [31]. In patients with MCI due to AD, but not cognitively stable MCI, elevated MMP-10 was accompanied by increased levels of other inflammatory proteins, suggesting that acceleration of cognitive decline is likely due to the cumulative effect of different pathological processes. One of these processes may be vascular pathology. Erhardt et al. showed that patients with mixed AD and vascular dementia show the highest value of CSF MMP-10, ptau, and other inflammatory markers, suggesting that vascular pathology may accelerate cognitive decline, possibly as a result of a disrupted blood-brain barrier and neuroinflammation [34]. Together, these studies suggest that elevated MMP-10 may be an early feature of AD, with potential diagnostic and prognostic value, especially if used in combination with ptau and other CSF markers of inflammation. Our study showed an increase in MMP-10 with the likelihood of AD as defined by PLM score and significantly higher levels of MMP-10 in T+ and N+ participants confirming previous findings from well-controlled clinical cohorts and validating the usefulness of this marker in a mixed memory clinic population. Collectively these data support that MMP-10 can be a useful biomarker of neurodegeneration, presumed due to AD. Elevated levels of MMP-10 have also been reported in other forms of dementia. For example, Boström et al. show increased CSF MMP-10 in patients with FTD, when compared to healthy controls [9]. Santaella et al. showed that elevated levels of CSF MMP-10 correlate with disease progression in patients diagnosed with Parkinson’s disease [35]. Despite these similarities with AD, no significant correlation was found with other inflammatory proteins, especially in FTD where decreased levels of inflammatory proteins are reported especially for proteins related to T cell function [9]. These studies indicate that cognitive decline in FTD and PD may be due to different pathological processes, when compared to AD, and further studies will be required to develop a composite inflammatory biomarker to distinguish different types of neurodegeneration.

In our study, ADA – an enzyme of purine metabolism – displayed the strongest correlation to ptau and total tau (with rho 0.6 and 0.619, respectively). ADA is a marker of cell mediated immunity. This enzyme degrades adenosine, which has an anti-inflammatory function, resulting in increased inflammation [36]. Through the metabolism of adenosine, ADA impacts sleep homeostasis [37, 38]. Increased levels of ADA have been detected in blood samples from patients with chronic insomnia [39]. Sleep disturbance is a well-recognized risk factor for dementia, and individuals with sleep problems have a risk ratio of 1.6 for developing AD compared to individuals with no sleep disturbance [40]. To our knowledge, our study is the first to report increased levels of ADA in CSF of individuals with neurodegeneration presumed due to AD, while ADA activity was found to be increased in the temporal cortex of postmortem tissue from individuals at early-stage disease [41]. ADA has also been proposed as a biomarker and possible therapeutic target for a number of inflammatory conditions, including rheumatoid arthritis, cardiovascular disease, inflammatory bowel disease, and bacterial infection [42–45]. Further studies are required to elucidate the potential role for ADA in the diagnosis and treatment of AD. Our study observed an increase of inflammation markers with the likelihood of AD as expressed by the PLM class, illustrating the impact of inflammation on neurodegeneration across the AD continuum. These observations indicate that markers of inflammation may provide a useful adjunct to established AD biomarkers to help improve the assessment of active neurodegeneration. In this context, ADA showed a significant step change from very low and low (PLM Class 0,1) to high and very high (PLM Class 2,3) likelihood of AD. With an AUROC of 0.788, our results indicate that CSF levels of ADA could be a useful predictor for neurodegeneration supporting risk-based patient stratification to help identify individuals who would benefit from additional tests to establish a diagnosis. Analysis of CSF MMP-10 and HGF demonstrated a gradually increased likelihood of AD, which allows differentiation between the very low and low likelihood stages.

Our study found that multiple functional pathways, including MAPK cascade and inflammatory response, were associated with elevated ptau and total tau values. Cullen et al. analyzed CSF samples from the BioFINDER study and found that these inflammatory pathways were altered in patients with AD. They used changes in inflammatory markers to predict an InflammAGE score representing the difference between an individual’s inflammatory and chronological age [46]. Their findings help elucidate the role of inflammation in healthy aging and neurodegeneration. Here, we demonstrate this in a mixed memory clinic patient cohort, suggesting utility in the future use of arrays of biomarkers representing functional pathways in the diagnosis, disease-stratification and prognosis of neurodegeneration.

Limitations worth noting: The sample size in our study is relatively small (105 subjects) and is derived from a heterogenous clinical population. For the reasons discussed earlier, no exclusion criteria were applied, and we cannot exclude the influence of mixed pathology, for example due to vascular pathology co-existing with AD. However, this limitation is typical of clinical practice and our approach provides a real-world test, where, despite the small numbers, inflammation markers gave robust signals irrespective of the potential for mixed disease and clinical heterogeneity. Markers including HGF, MMP-10, ADA, and TWEAK showed significant increase with ptau and total tau pathology and a gradual significant increase with PLM class demonstrating potential for translation into clinical application for evaluating the likelihood of neurodegeneration. We did not classify this cohort into diagnostic subgroups across the AD clinical spectrum. Instead, we focused on the established biological AD markers: Aβ_42_, ptau, and total tau to provide objective measures of pathology. The data collected in this study is cross-sectional and further studies would benefit from using longitudinal clinical data to further evaluate the ability of inflammation markers to assess the likelihood of conversion from MCI to AD. The seminal study by Boström et al. identified that 11 different inflammatory markers, including MMP-10, TWEAK, and HGF, have increased concentration in patients with MCI/AD compared to stable MCI preparing the ground for future use of inflammatory markers in prediction of progression risk to dementia [9].

### Conclusion

In this study, inflammation levels increased with presence of tau pathology but not with Aβ in a clinical cohort. These findings suggest a role for inflammation in the pathogenesis and progression of neurodegeneration. Despite sample heterogeneity, the concentration of markers such as HGF, MMP-10, ADA, and TWEAK correlates with increasing likelihood of AD showing potential for clinical translation. Further work is required to assess the prognostic efficacy of these markers when used in conjunction with established CSF measures of Aβ and tau in the clinical evaluation of neurodegeneration.

## Supplementary Material

Supplementary Material

## Data Availability

Due to research governance and ethical considerations, supporting data cannot be made openly available. Bona fide researchers may request supporting data by making a formal application to the corresponding author. The investigators will provide the data to any interested party subject to ethical approval. For the purpose of open access, the authors have applied a Creative Commons Attribution (CC BY) license to any Author Accepted Manuscript version arising.
