# Supplementary material for: Biomarkers of Inflammation Increase with Tau and Neurodegeneration but not with Amyloid-β in a Heterogenous Clinical Cohort
Source: J Alzheimers Dis. 2022 Oct 11;89(4):1303–14. doi: 10.3233/JAD-220523 (PMC9661320; doi:10.3233/JAD-220523)
Supplement: Supplementary Material [file jad-89-jad220523-s001.pdf]

Supplementary Material

Biomarkers of Inflammation Increase with Tau and Neurodegeneration but not with Amyloid-β in a Heterogenous Clinical Cohort

Supplementary Table 1. Abbreviations, full names, and functional pathways for the 52 inflammation markers presented in this study

Abbreviation	Full Name	Functional Pathway
4E-BP1	Eukaryotic translation initiation factor 4E-binding protein	
ADA	Adenosine Deaminase	1,2,3,7,9,10,11
CCL11	Eotaxin-1	3,4,5,8
CCL19	C-C motif chemokine 19	1,2,3,4,5,7,8,9,11
CCL23	C-C motif chemokine 23	4,5,7,8,
CCL25	C-C motif chemokine 25	3,4,5,8
CCL3	Macrophage inflammatory protein 1-alpha	1,2,4,5,7,8,11
CCL4	C-C motif chemokine 4	3,4,5,7,8
CD40	CD40L receptor	1,2,4,7,8,9,11
CD5	T-cell surface glycoprotein CD5	1,3
CD8A	T-cell surface glycoprotein CD8 alpha chain	3,9
CDCP1	CUB domain-containing protein 1	
CSF-1	Macrophage colony-stimulating factor 1	3,4,5,7
CST5	Cystatin D	
CX3CL1	Fractalkine	1,3,4,7,8,11
CXCL1	C-X-C motif chemokine 1	2,4,5,7
CXCL10	C-X-C motif chemokine 10	4,5,7
CXCL11	C-X-C motif chemokine 11	4,5,8
CXCL5	C-X-C motif chemokine 5	4,5,9
CXCL6	C-X-C motif chemokine 6	4,5,10
CXCL9	C-X-C motif chemokine 9	4,5,11
DNER	Delta and Notch-like epidermal growth factor related receptor	
FGF-19	Fibroblast growth factor 19	9
FGF-5	Fibroblast growth factor 5	
Flt3L	Fms-related tyrosine kinase 3 ligand	1,4,8
HGF	Hepatocyte growth factor	1,4,5,7,8,11
IL-10RB	Interleukin-10 receptor subunit beta	4,7
IL-12B	Interleukin-12 subunit beta	2,3,4,7,9
IL-18R1	Interleukin-18 receptor 1	2,3,7,8
IL-1b	Interleukin-1 subunit beta	
IL-2	Interleukin-2	1,2,3,4,7,8,9,11
IL-18	Interleukin-18	8
IL-6	Interleukin-6	1,2,3,4,5,7,8,9,11
IL-8	Interleukin-8	3,4,5,7
LAP TGF-beta1	Latency-associated peptide transforming growth factor Beta	1,2,3,4,5,6,7,8,9,10,11

LIF-R	Leukemia inhibitory factor receptor	4,11
MCP-1	Monocyte chemotactic protein 1	1,3,4,5,8
MCP-2	Monocyte chemotactic protein 2	4,5,7,8,11
MCP-4	Monocyte chemotactic protein 4	4,5,7,8
MMP-1	Matrix metalloproteinase-1	4,6,7
MMP-10	Matrix metalloproteinase-10	6
OPG	Osteoprotegerin	
PD-L1	Programmed cell death 1 ligand 1	1,3,9,11
SCF	Stem cell factor	3,8
TGF-alpha	Transforming growth factor alpha	8
TNFB	TNF-beta	4,7,9,10
TNFRSF9	Tumor necrosis factor ligand superfamily member 9	4.7.11
TNFSF14	Tumor necrosis factor ligand superfamily member 14	1,3,4,5
TRAIL	TNF-related apoptosis-inducing ligand	1
TWEAK	Tumor necrosis factor (Ligand) superfamily, member 12	1,3
uPA	Urokinase-type plasminogen activator	2,3,5,10,11
VEGFA	Vascular endothelial growth factor A	1,3,4,5,8,10,11

Functional Pathways: 1. Apoptosis, 2. Cell Activation Immune Response, 3. Cell Adhesion, 4. Cell Response to Cytokine, 5. Chemotaxis, 6. Extracellular Matrix Organisation, 7. Inflammatory Response, 8. MAPK Cascade, 9. Regulation of immune response, 10. Response to Hypoxia, 11. Secretion
